# The effect of music therapy on cognitive functions in patients with Alzheimer’s disease: a systematic review of randomized controlled trials

**DOI:** 10.1186/s13195-023-01214-9

**Published:** 2023-03-27

**Authors:** Malak Bleibel, Ali El Cheikh, Najwane Said Sadier, Linda Abou-Abbas

**Affiliations:** 1 Faculty of Medical Sciences, Neuroscience Research Centre, Lebanese University, Beirut, Lebanon; 2 Pierre and Marie Curie Campus, Sorbonne University, Paris, France; 3 College of Health Sciences, Abu Dhabi University, Abu Dhabi, United Arab Emirates; 4 INSPECT-LB (Institut National de Santé Publique Epidémiologie Clinique Et Toxicologie-Liban), Beirut, Lebanon

**Keywords:** Alzheimer’s disease, AD, Cognitive functions, Music therapy, Music intervention

## Abstract

**Background:**

The use of music interventions as a non-pharmacological therapy to improve cognitive and behavioral symptoms in Alzheimer’s disease (AD) patients has gained popularity in recent years, but the evidence for their effectiveness remains inconsistent.

**Objectives:**

To summarize the evidence of the effect of music therapy (alone or in combination with pharmacological therapies) on cognitive functions in AD patients compared to those without the intervention.

**Methods:**

A systematic literature search was performed in PubMed, Cochrane library, and HINARI for papers published from 1 January 2012 to 25 June 2022. All randomized controlled trials that compared music therapy with standard care or other non-musical intervention and evaluation of cognitive functions are included. Cognitive outcomes included: global cognition, memory, language, speed of information processing, verbal fluency, and attention. Quality assessment and narrative synthesis of the studies were performed.

**Results:**

A total of 8 studies out of 144 met the inclusion criteria (689 participants, mean age range 60.47–87.1). Of the total studies, 4 were conducted in Europe (2 in France, 2 in Spain), 3 in Asia (2 in China, 1 in Japan), and 1 in the USA. Quality assessment of the retrieved studies revealed that 6 out of 8 studies were of high quality. The results showed that compared to different control groups, there is an improvement in cognitive functions after music therapy application. A greater effect was shown when patients are involved in the music making when using active music intervention (AMI).

**Conclusion:**

The results of this review highlight the potential benefits of music therapy as a complementary treatment option for individuals with AD and the importance of continued investigation in this field. More research is needed to fully understand the effects of music therapy, to determine the optimal intervention strategy, and to assess the long-term effects of music therapy on cognitive functions.

## Introduction

Alzheimer’s disease (AD) is a progressive, incurable neurological illness that is the most common cause of dementia, affecting an estimated 5% of men and 6% of women over the age of 60 worldwide [1]. The prevalence of AD increases exponentially with age, with 1% of those aged 60 to 64 years old and 24% to 33% of those aged 85 years or older affected [2]. As the global population ages, it is anticipated that the number of individuals with Alzheimer’s disease will increase.

Neuropsychiatric symptoms, such as apathy, depression, and agitation, are commonly observed in individuals with AD, in addition to the more well-known cognitive symptoms such as memory loss, visuospatial problems, and difficulties with executive functions [3, 4]. These symptoms can cause a significant burden to patients, caregivers, and society as a whole [5]. While pharmacological therapies have been used to manage these symptoms, they have not always been effective in achieving long-term clinical efficacy [6]. As a result, non-pharmacological interventions have gained increasing attention as a complementary treatment option for managing neuropsychiatric symptoms in AD. Such therapies include cognitive training and music therapy which have been used for decades to improve symptoms of dementia [7].

Music Therapy is the use of music to address the physical, emotional, cognitive, and social needs of individuals [8]. The American Music Therapy Association describes music therapy as the use of music interventions in a clinical and evidence-based manner to achieve specific goals, which are tailored to the individual, by a professional who is credentialed and has completed an approved music therapy program [8]. Music therapy incorporates a crucial aspect of the interaction between the client and therapist through an evidence-based model [9]. It can include both active techniques, such as improvisation, singing, clapping, or dancing, and receptive techniques, where the client listens to music with the intention of identifying its emotional content [9]. In music listening approaches, the therapist creates a personalized playlist for the client, which can either be an individualized program or chosen by the therapist [9, 10]. Generalized music interventions use music without a therapist present, with the goal of enhancing the patient’s well-being, and can include both active and music listening protocols. Music listening is used to stimulate memories, verbalization, or encourage relaxation [9].

For many years, music therapy has been used to help manage symptoms of dementia [9, 11]. Music therapy can improve mood, cognitive functions, memory, and provide a sense of connection and socialization for patients who may be isolated [12, 13]. Studies have found that musical training may help mitigate the effects of age-related cognitive impairments, and the capacity of persons to remember music makes it a good stimulus that engages AD patients [7, 14, 15]. After listening to music, AD patients showed improvement in categorical word fluency [16], autobiographical memory [17, 18], and the memory of the lyrics [15]. Additionally, it can provide an opportunity for caregivers to participate in therapy sessions, which can improve the overall caregiving experience by giving them the opportunity for self-expression allowing them to depict their thoughts and emotions [19].

The specific mechanisms by which music therapy is beneficial are not fully understood. In 2003, research indicates that music may activate neural networks that remain intact in individuals with AD [20]. A recent study by Jacobsen et al. [21] used 7 T functional magnetic resonance imaging to examine the brain’s response to music and identify regions involved in encoding long-term musical memory. When these regions were evaluated for Alzheimer’s biomarkers, such as amyloid accumulation, hypometabolism, and cortical atrophy, the results showed that, although amyloid disposition was not significantly lower in the AD group compared to the control group, there was a substantial reduction in cortical atrophy and glucose metabolism disruption in AD patients [21]. These findings suggest that musical memory regions are largely spared and well-preserved in AD, which could help explain why music therapy is so effective in retrieving verbal and musical memories in individuals with the disease [21].

One experimental paradigm used to study the effects of music therapy in AD is the use of live music performances, in which a music therapist plays live music for individuals with the disease in a group setting [22]. Another paradigm is the use of individualized music, in which a music therapist creates a playlist of personalized music for an individual with the disease to listen to at home [23]. Both paradigms have been shown to be effective in improving mood and reducing agitation in individuals with AD [22, 23].

The advantages of music therapy for AD patients include its non-invasive nature and lack of side effects, its ability to address multiple symptoms at once, and its cost-effectiveness and ease of implementation [9, 18, 24, 25]. However, there are also some limitations to its application. Music therapy may not be suitable for patients with severe dementia [26] as their cognitive and physical abilities may be too impaired to fully participate in therapy sessions. Additionally, it requires trained therapists [8, 9], who may not be easily accessible in some areas. In this review, we aimed to summarize the evidence of the effect of music therapy (alone or in combination with pharmacological therapies) on cognitive functions in AD patients compared to those without the intervention.

## Methods

This systematic review was performed following the Preferred Reporting Items for Systematic Reviews and Meta-Analysis (PRISMA 2009) guidelines [27]. The protocol of this study was registered in PROSPERO. A statement of ethics was not required.

We used the PICO framework (population, intervention, comparator, and outcome) as follows:

- P: Alzheimer patients
- I: Music therapy (alone or in combination with pharmacological therapies)
- C: Alzheimer patients with and without the intervention
- O: Cognitive functions

### Search strategy and databases

A systematic literature search of PubMed, Cochrane, and HINARI was performed for studies published in peer-reviewed journals from 1 January 2012 up to 25 June 2022. The databases were searched using the keywords of “Alzheimer’s Disease,” “AD,” “music therapy,” “music intervention,” “cognitive functions,” and “cognition.” Keywords were combined using the Boolean operators “OR” and “AND.”

### Study selection and eligibility criteria

All randomized controlled trials (RCTs) published between 2012 and 2022 in the English language and providing quantitative measures of the association between AD and music therapy and its effect on cognitive functions were included in our review. Studies that assess the effect of music therapy on patients with a probable diagnosis of AD or studies where the music therapy was combined with another non-pharmacological therapy are excluded.

### Data extraction

Search and identification of eligibility according to inclusion criteria and extraction of data were performed by the two reviewers MB and AC. For each paper, detailed information was collected on: study information (author’s name, publication year, and location), sample characteristics (sample size, age, and gender), study design, intervention details (description, duration) the control group, and the cognitive outcome measures.

### Methodological quality assessment

A methodological quality assessment of all included studies was performed by two independent reviewers (MB and AC) using the Jadad scale for RCTs [28]. Although not used as a criterion for study inclusion or exclusion. Jadad scale is developed to assess randomized controlled trials on the bases of 3 essential items: (1) randomization, 1 point if randomization is mentioned 1 additional point if the method of randomization is appropriate and deduct 1 point if the method of randomization is inappropriate,(2) blinding 1 point if blinding is mentioned, 1 additional point if the method of blinding is appropriate, deduct 1 point if the method of blinding is inappropriate; (3) an account of all patients, the fate of all patients in the trial is known. If there are no data, the reason is stated. It is commonly considered that a study is of “high quality” if it scores 3 points or more.

## Results

### Study selection

**Fig. 1:**
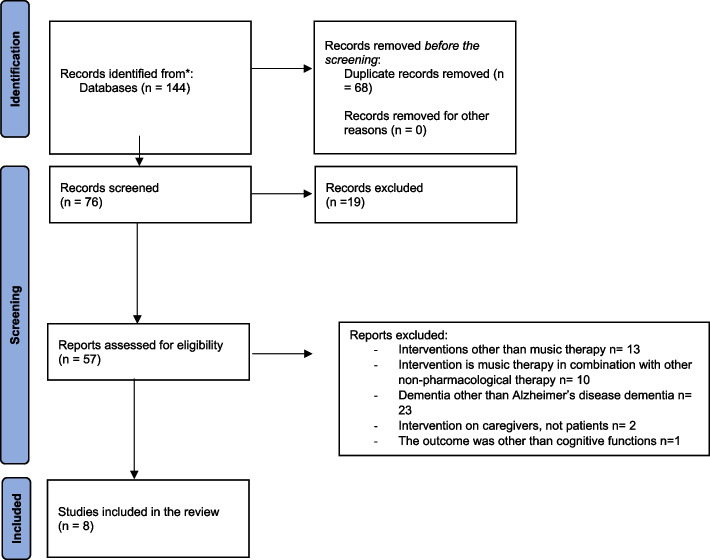
PRISMA flow diagram of the selection procedure

The flowchart of the study selection process is presented in Fig. 1. The literature search identified a total of 144 records. After the exclusion of duplicate records and non-relevant abstracts, 57 studies were retained. After reviewing the full text, 49 studies were excluded according to our inclusion and exclusion criteria. In the end, a total of 8 full-text studies were included in the qualitative synthesis.

### Study characteristics

Characteristics of included studies are presented in Table 1. The final sample was composed of 8 RCTs, 4 studies were conducted in Europe (2 in France, 2 in Spain), 3 studies in Asia (2 in China, 1 in Japan), and one in the USA. All these studies were published in the English language in peer-reviewed journals. Included trials showed a total of 689 participants (300 females, 43.54%). Sample sizes ranged from 39 [29] to 298 [30]. Mean ages ranged from 60.47 [31] to 87.1 [26]. Participants’ stages of AD dementia varied from mild to severe. Mean Mini-Mental State Examination (MMSE) [32] at baseline is assessed in 7 trials out of 8 and varied from 4.65 [29] to 25.07 [33].

**Table 1:** Characteristics and main findings of studies included in the systematic review

Study (author, year, country)	Study design	Number of participants	Sex	Mean age	MMSE, mean score at baseline	Description of intervention	Total number of sessions	Duration	Control group	Cognitive outcome measures
[29] Japan	RCT	39	21 females	79.25	4.65	AMI: Listen to music played on a CD player and clap, sing, or dance RMI: Participants listen to music played on a CD player	10	10 weeks	Standard	Short-term: Faces Scale (***p*-value < 0.01)** Long-term: BEHAVE-AD (***p*-value < 0.025) **
[26] France	*RCT*	48	32 females	87.1	10.2	AMI and RMI: Listening and participating by signing and/or by using percussion instruments to accompany the musical track	8	4 weeks	Cooking sessions	Cognitive abilities (SIB) **(*p*-value = 0.2) **
[34] Spain	RCT	42	28 females	77.5	15.02	AMI and RMI: Listening to the music played on a high-quality stereo and singing, moving arms and hands, or guessing the name of it	12	6 weeks	Standard	Global cognition (MMSE) **(*p*-value < 0.001) **
[33] France	RCT	59	23 females	78.8	25.07	AMI: Patients should sing previously chosen songs according to their preferences	12	3 months	Painting intervention	Verbal memory (FCRT) (***p*-value = 0.001)**, speed of information-processing and mental flexibility (TMT) (***p*-value = 0.12)** working memory (symbol test and digit span test) **(*p*-value = 0.001)**, speed of information-processing and inhibition (Stroop test) **(*p*-value = 0.03)**, mental flexibility and verbal fluency (Letter and Category fluency tests) **(*p*-value = 0.23/*p*-value = 0.37)**, cognitive dysexecutive syndrome (FAB) **(*p*-value = 0.37) **
[31] USA	RCT	53	46 females	60.47	N/A	RMI: Listening to 12 min of relaxing music of their choice	N/A	12 weeks	Kritan Kriya meditation program	Memory functioning (MFQ) **(*p*-value = 0.007)**, executive function (TMT) **(*p*-value = 0.006)**, psychomotor speed, attention, and working memory (DSST) (***p*-value = 0.04) **
[30] China	RCT	298	57 females	69.7	13.26	AMI: Singing their familiar and favorite songs and if they fail they just listen (RMI)	N/A	3 months	Standard	Global cognition (MMSE), short-term and long-term memory (WHO-UCLA AVLT), language function (semantic verbal fluency test) **(*p*-value < 0.05) **
[35] China	RCT	60	38 females	69.8	17.63	RMI: Listening to songs chosen according to the patient’s pathogenic condition, education level, and personal preferences	N/A	3 months	Standard	Global cognition **(MMSE and MoCA)** (MMSE**: *p*-value = 0.003**, MoCA ***p*-value = 0.000**)
[36] Spain	RCT	90	55 females	80.87	18.035	AMI: Participants keep rhythm by clapping hands, dancing or music quiz RMI: Participants listen to music played on a CD player	12	3 months	Watching nature videos	Global cognition (MMSE) (AMI: *p*-value < 0.001 RMI: *p*-value < 0.001)

*AMI* active music intervention, *RMI* receptive music intervention, *MMSE* Mini-Mental State Examination, *FCRT* Free and Cued Recall Test, *TMT* Trail Making Test, *FAB* frontal assessment battery, *WHO-UCLA AVLT* World Health Organization University of California-Los Angeles Auditory Verbal Learning test, *SIB* severe impairment battery, *MFQ* Memory Functioning Questionnaire, *DSST* Digit Symbol Substitution Test, *MoCA* Montreal Cognitive Assessment

### Intervention characteristics

#### Music therapy approach

Music therapy methods were heterogeneous across the included studies. In one study, the active music therapy approach used was singing with the played songs [33]. Two other studies used the receptive (passive) music therapy approach which consists in listening to music and songs played on a CD player [31, 35]. The remaining five studies were based on a combination of both active and receptive music approaches [26, 29, 30, 34, 36].

#### Comparators

In four studies, music therapy intervention was compared to standard care [29, 30, 34–36], while in the four remaining studies, different interventions other than music therapy were used as comparators such as: watching nature videos [36], painting [33], cooking [26], and practicing meditation [31].

#### Application of the intervention

Only three trials were conducted by a music therapist [29, 34, 36], 1 trial was conducted by a professional choir conductor [33], 1 by musicians [30] and the 3 remaining trials were conducted with facilitators with no musical expertise [26, 31, 35].

#### Types of applied music

Seven trials out of 8 were based on individualized songs (chosen according to patient’s preferences or songs that are used to evoke positive emotions in them) [29–31, 33–36]. The remaining trial was based on familiar songs chosen without considering the patient’s preferences [26].

### Outcome characteristics

The included studies assessed different outcomes, but we focused on domains directly related to outcome inclusion criteria: global cognition, memory, language, speed of information processing, verbal fluency, and attention. All cognitive outcomes and measurement tools used across studies are listed in Table 1.

### Risk of bias

The quality of trials was assessed by Jadad scales [28]. Studies with scores ≥ 3 were classified as high-quality studies and those of ≤ 2 were classified as “low-quality” studies. [26, 29–31, 33, 36] studies were considered high-quality studies while [34, 35] studies were considered of low-quality. Blinding of participants was not possible due to the nature of the intervention considered in this review. Randomization was mentioned in all studies except one study [34]. Results of the quality assessment of all studies using the Jadad scales are summarized in Table 2.

**Table 2:** Quality assessment of the retrieved studies using Jadad score items

References	Randomization mentioned	Appropriateness of randomization	Blinding mentioned	Appropriateness of blinding	An account of all participants or a description of withdrawals or dropouts	Total
[29]	1	1	1	0	1	4/5
[26]	1	0	1	0	1	3/5
[34]	0	0	1	0	0	1/5
[33]	1	1	1	0	1	4/5
[31]	1	1	1	0	1	4/5
[30]	1	1	1	0	1	4/5
[35]	1	1	0	0	0	2/5
[36]	1	1	1	0	0	3/5

### Results of individual studies

Sakamoto et al. [29] studied the effect of music intervention (active and passive) on patients with severe dementia. Results showed that there is a short-term improvement in emotional state assessed by the facial scale which is a tool commonly used by psychologists and healthcare professionals to assess and code facial expressions, both positive and negative, to determine a patient’s emotional state [37, 38]. In addition to eliciting positive emotions, music therapy has been shown to have long-term benefits in reducing behavioral and psychological symptoms of dementia assessed by the Behavioral Pathology in Alzheimer’s Disease (BEHAVE-AD) Rating Scale, a well-established instrument to assess and evaluate behavioral symptoms in AD patients, as well as to evaluate treatment outcomes and identify potentially remediable symptoms [39].

The study by Narme et al. [26] was conducted to evaluate the effectiveness of music and cooking interventions in improving the emotional, cognitive, and behavioral well-being of AD and mixed dementia patients. The study lasted 4 weeks and involved 48 patients, who received two 1-h sessions of either music or cooking interventions per week. Both interventions showed positive results, such as improved emotional state and reduced the severity of behavioral disorders, as well as reduced caregiver distress. However, there was no improvement in the cognitive status of the patients. Although the study did not find any specific benefits of music interventions, it suggests that these non-pharmacological treatments can improve the quality of life for patients with moderate to severe dementia and help to ease caregiver stress [26].

The study by Gómez Gallego and Gómez García [34] showed a significant increase in MMSE scores, especially in the domains of orientation, language and memory [34]. Subsequent study from the same author aiming to compare the benefits from active music therapy versus receptive music therapy or usual care on 90 AD patients showed impressive results of active music intervention improving cognitive deficits and behavioral symptoms [36]. Other supportive data revealed an increase of MMSE and MoCA scores over the study duration in the intervention group, in comparison to the control group [35].

The study by Pongan et al. [33] examined the effects of singing versus painting on 50 AD patients over a period of 12 weeks. Results showed that both therapies elicited benefits in reducing depression, anxiety, and pain. The only advantage that the singing group had over the painting group is the stabilization of verbal memory (assessed using FCRT) over time [33].

Lyu et al. [30] study aimed to investigate the effects of music therapy on cognitive functions and mental well-being in AD patients. The study utilized the World Health Organization University of California-Los Angeles Auditory Verbal Learning Test (WHO-UCLA AVLT) to assess the short-term and long-term memory of the participants. Subjects were tested on their ability to recall 15 verbal words immediately and after a delay of 30 min. The results showed that music therapy was more effective in improving verbal fluency and alleviating psychiatric symptoms and caregiver distress than lyric reading in AD patients. The stratified analysis revealed that music therapy improved memory and language ability in mild AD patients and reduced psychiatric symptoms (delusions, hallucinations, agitation/aggression, dysphoria, anxiety, euphoria, apathy, disinhibition, irritability/lability, and aberrant motor activity) and caregiver distress in moderate or severe AD patients. However, no significant effect was found on daily activities in any group of patients [30].

Innes et al. [31] study consisted of testing music listening therapy over a period of 12 weeks. Cognitive functions were assessed through various measures, including memory (using the Memory Functioning Questionnaire MFQ), executive function (using the Trail Making Test (TMT) Parts A and B), and psychomotor speed, attention, and working memory (using the 90-s Wechsler Digit-Symbol Substitution Test). The scores assessed at baseline, 3 months, and 6 months after therapy showed an improvement in measures of memory function, psychological status, and cognitive performance including executive functions, working memory, processing speed, and attention [31].

## Discussion

Neurodegenerative diseases, such as dementia, pose a major challenge to global health and will continue to increase in impact with the aging population. AD is a widespread form of dementia affecting a large number of elderly individuals globally and may contribute to 60–70% of cases [40]. Despite efforts to find effective treatments through pharmacological means, the results have been disappointing in recent decades. As a result, non-pharmacological therapies have gained more attention as a way to improve cognitive, behavioral, social, and emotional functions in AD patients.

Music therapy has been shown to induce plastic changes in some brain networks [41], facilitate brain recovery processes, modulate emotions, and promote social communication [42], making it a promising rehabilitation approach. Thus, the present systematic review aimed to systematically synthesize the impact of music therapy on cognitive functions in AD patients. Out of the eight studies reviewed, totaling 689 subjects, seven studies found a significant and positive effect of music therapy on enhancing cognitive functions in individuals with AD. However, one study by Narme et al. [26] did not find evidence of the efficacy of music therapy on cognitive functions [26]. This result may be due to the use of music that was chosen by the therapist, rather than being based on the patient’s preferences, and the use of cooking as a control group rather than a standard group to test the efficacy of the intervention. Furthermore, Narme et al. [26] suggested that a larger sample size would be beneficial in conducting parametric analysis, which could provide more robust results [26]. These findings highlight the potential benefits of music therapy as a non-pharmacological intervention for AD patients.

Six out of eight studies revealed that patients who underwent Active Music Intervention (AMI) had better outcomes compared to those who underwent Receptive Music Intervention (RMI) [29, 30, 33–36]. On the other hand, the findings of the studies by Innes et al. [31] and Wang et al. [35] that used only the RMI approach, showed a positive impact on cognitive functions in AD patients [31, 35].

In the study by Innes et al. [31], both the meditation and music listening groups showed significant improvements in cognitive functions, without a significant difference between the two groups. In the study by Wang et al. [35], music therapy was found to be an effective adjuvant to support pharmacological interventions in AD, leading to significant improvements in the MMSE and MoCA scores. It is worth noting that AMI and RMI differ in terms of the level of patient involvement and the objectives of the therapy. AMI involves the direct participation of patients in musical activities such as singing, playing an instrument, or moving to the beat, whereas RMI consists of passive listening to music. From a functional and physiological perspective, AMI may have a greater impact on cognitive and emotional processes due to the increased level of engagement and interaction with the music [36]. AMI has been shown to activate brain regions involved in auditory processing, motor control, and emotional regulation, leading to improved cognitive functions and reduced agitation and anxiety [41]. On the other hand, RMI may have a more relaxing effect, as it can induce changes in heart rate and breathing, reducing stress levels and improving sleep quality [42]. Based on our systematic review, it is not possible to draw conclusions about the optimal music types (classic music, familiar songs, individualized songs…) for music therapy in patients with AD. This is due to the heterogeneity of the studies included in our review, including differences in the types of music used and the methods of exposure. Therefore, it is not possible to determine with certainty which type of music is most effective for improving cognitive functions in AD patients. Further research is needed to establish the optimal music types and optimal duration of music therapy in this population. Our findings also revealed that individualized music playlists, consisting of songs chosen based on the patient’s preferences, showed improvement in cognitive functions, particularly in memory. A study by [31] used relaxing music in the intervention group, chosen according to patients’ preferences. The music listening CD to be heard by patients in this study contained selections from Bach, Beethoven, Debussy, Mozart, Pachelbel, and Vivaldi, which resulted in an improvement in cognitive functions. This is consistent with the [43] study which showed that listening to classical music, specifically selections from Mozart, could result in a temporary improvement in certain cognitive tasks such as abstract/spatial reasoning tests. While the “Mozart Effect” has been linked more to the acute arousal brought on by the pleasure of listening to music, rather than a direct impact on cognitive ability [44], both studies highlight the potential for listening to classical music to have a positive impact on cognitive functions.

The improvement in orientation, language, and memory domains in individuals with AD, as reported in the studies by [34, 36], can be attributed to several factors such as the use of an individualized playlist or the presence of a music therapist to perform the sessions. The study by [30] suggests that music intervention has a positive effect on verbal fluency, memory, and language in individuals with AD. The rhythmic and repetitive elements of music regulate brain function, and musical activities such as singing and playing instruments can activate neural networks involved in memory and language processing.

Further beneficial effects other than improved cognitive behaviors, memory, language, and orientation, the study by [29] showed a positive impact on the emotional state of the patients. This is consistent with the idea that several cognitive processes such as perception, attention, learning, memory, reasoning, and problem-solving, are all influenced by emotions [45]. However, the positive effects observed in the emotional state of the patients disappeared 3 weeks after the intervention period. The effects of the intervention lasted after the follow-up for a period that varied between studies [29–31, 33, 35], from 1 month [33] to 6 months [30, 31]. Further research is needed to determine the most effective and optimal duration for music therapy interventions.

Our review has some limitations including differences in participant characteristics (participant age/severity of illness/cognitive ability…), outcome measures, and intervention methods, that may have influenced the results. Additionally, the music therapy interventions used in the studies differed, with activities ranging from singing to playing instruments. These factors, combined with the small number of studies included in the review, limit the power of our findings. Furthermore, the heterogeneity of the interventions and outcome measures used in the studies makes it difficult to perform a meta-analysis and combine the data in a meaningful way. The varying methods of music selection and exposure also pose challenges in synthesizing the results.

## Conclusion

The findings of this review suggest that music therapy could have a positive impact on cognitive functions in patients with AD. This supports the growing body of evidence that targets music therapy as a promising cognitive rehabilitating process aiming to improve cognitive functions in individuals with AD dementia like memory, executive functions, or attention. Improvements in these cognitive functions can, in turn, enhance the quality of life of both the patients and their caregivers. However, more research is needed to fully understand the mechanisms behind these effects and to determine the optimal approach to music therapy for this population, including the time frame for follow-up evaluations. Nevertheless, the results of this review highlight the potential benefits of music therapy as a treatment option for individuals with AD and the importance of continued investigation in this field, including long-term follow-up assessments to determine the sustained impact of music therapy on cognitive functions.

## Data Availability

All data generated or analyzed during this study are included in this published article.

## Abbreviations

AD: Alzheimer’s disease
AMI: Active music therapy
BEHAVE-AD: Behavior Pathology in Alzheimer’s Disease
DSST: Digit Symbol Substitution Test
FAB: Frontal assessment battery
FCRT: Free and Cued Recall Test
MFQ: Memory Functioning Questionnaire
MMSE: Mini-Mental State Examination
MoCA: Montreal Cognitive Assessment
PET: Positron emission tomography
PRISMA: Preferred Reporting Items for Systematic Reviews and Meta-Analysis
RCTs: Randomized controlled trials
RMI: Receptive music therapy
SIB: Severe impairment battery
TMT: Trail Making Test
USA: United States of America
WHO-UCLA AVLT: World Health Organization University of California-Los Angeles Auditory Verbal Learning test
